# State of the art in nail dosimetry: free radicals identification and reaction mechanisms

**DOI:** 10.1007/s00411-014-0512-2

**Published:** 2014-01-28

**Authors:** F. Trompier, A. Romanyukha, R. Reyes, H. Vezin, F. Queinnec, D. Gourier

**Affiliations:** 1 Institut de Radioprotection et de Sûreté Nucléaire (IRSN), Fontenay-aux-Roses, France; 2 Naval Dosimetry Center, US Navy, Bethesda, MD USA; 3 Uniformed Services University of the Health Sciences, Bethesda, MD USA; 4 Laboratoire de Spectrochimie Infrarouge et Raman (LASIR), Univertisy of Lille, Lille, France; 5 Laboratoire de Chimie de la Matière Condensée de Paris, CNRS-UMR 7574, Ecole Nationale Supérieure de Chimie de Paris, Paris, France

**Keywords:** EPR spectroscopy, Dosimetry, Human nails, Radiological accident, Q- and X-bands

## Abstract

Until very recently, analysis of bone biopsies by means of the method of electron paramagnetic resonance (EPR) collected after surgery or amputation has been considered as the sole reliable method for radiation dose assessment in hands and feet. EPR measurements in finger- and toenail have been considered for accident dosimetry for a long time. Human nails are very attractive biophysical materials because they are easy to collect and pertinent to whole body irradiation. Information on the existence of a radiation-induced signal in human nails has been reported almost 25 years ago. However, no practical application of EPR dosimetry on nails is known to date because, from an EPR perspective, nails represent a very complex material. In addition to the radiation-induced signal (RIS), parasitic and intense signals are induced by the mechanical stress caused when collecting nail samples (mechanically induced signals—MIS). Moreover, it has been demonstrated that the RIS stability is strongly influenced not only by temperature but also by humidity. Most studies of human nails were carried out using conventional X-band microwave band (9 GHz). Higher frequency Q-band (37 GHz) provides higher spectral resolution which allows obtaining more detailed information on the nature of different radicals in human nails. Here, we present for the first time a complete description of the different EPR signals identified in nails including parasitic, intrinsic and RIS. EPR in both X- and Q-bands was used. Four different MIS signals and five different signals specific to irradiation with ionizing radiation have been identified. The most important outcome of this work is the identification of a stable RIS component. In contrast with other identified (unstable) RIS components, this component is thermally and time stable and not affected by the physical contact of fingernails with water. A detailed description of this signal is provided here. The discovery of stable radiation-induced radical(s) associated with the RIS component mentioned opens a way for broad application of EPR dosimetry in human nails. Consequently, several recent dosimetry assessments of real accident cases have been performed based on the described measurements and analyses of this component.

## Introduction

Electron paramagnetic resonance (EPR) spectroscopy is a versatile and key tool for dose assessment after a severe radiological accident using biological materials collected from victims (bones and tooth enamel) or other materials irradiated during the accident (sugars, glass from personal items, etc.). EPR spectroscopy is used in a multi-parametric approach together with biological dosimetry and other dose reconstruction tools, such as numerical simulation. Review of the available publications and significant experience of the Institut de Radioprotection et de Sûreté Nucléaire (IRSN) shows that EPR has been mainly used with bones biopsies, specifically for cases of highly localized accidental irradiation of extremities (Regulla and Deffner 1989; Schauer et al. 1996; Wu et al. 1998; Clairand et al. 2006; Trompier et al. 2007a; Clairand et al. 2008; IAEA 2004). As a matter of fact, in the case of a partial body irradiation (e.g., source handlings, radiotherapy and radiology accidents or sources in a person’s clothes’ pocket), the local dose can be several orders of magnitude higher than the whole body dose. Therefore, in such cases, subsequent assessment of the maximum dose is highly desirable for a competent choice of the therapeutic strategy (Tamarat et al. 2012; Benderitter et al. 2010; Bey et al. 2010). For some scenarios with a good knowledge of accident parameters, it is possible to assess the local dose using numerical simulations; however, in the case of sources-handling, which is a very common type of radiation accident, the applicability of numerical approaches is limited due to the difficulty in reconstructing the irradiation configuration (lack of details). Typically, in accidental scenarios, the dose distribution is characterized by high gradients. Before the appearance of any clinical signs, it is usually very difficult to determine the location of the hot spots of irradiation. EPR bone dosimetry cannot be easily applied in the early management phase of victims because of the invasiveness of sampling and is usually performed after amputation or using surgical residues of bone.

For these reasons, the possibility to evaluate dose on nails is highly attractive. Human nails were already considered several decades ago for radiation accident dosimetry (Dalgarno and McClymont 1989; Symons et al. 1995) and for triage in case of large scale events (2007). Nails are easy to collect and can possibly give an estimation of the dose distribution when nails from each finger or toe can be analyzed separately. More recently, large efforts have been made to understand the free radicals mechanism in nails and establish EPR nail dosimetry with different approaches (Romanyukha et al. 2007a, 2010; Reyes et al. 2008, 2009, 2012; Trompier et al. 2007b, 2009; Black and Swarts 2010; Wilcox et al. 2010; He et al. 2011). In spite of these efforts, until now, no practical application has been reported, to the best of the authors’ knowledge, mainly because several difficulties have been identified in the development of EPR dosimetry on nails. Chandra and Symon (1987) reported the presence of parasitic EPR signals induced by the mechanical stress when nails are cut, which overlap the radio-induced signals (RIS). Trompier et al. (2007b) reported the effect of humidity on the stability of both the RIS and the so called mechanically induced signals (MIS). The fading of the RIS and MIS is correlated with the level of humidity: the highest humidity level causes the fastest signals’ decay. As a consequence, hand washing can eliminate the RIS and MIS, at least those components of the RIS and MIS that were identified at that time. The dose estimation on nails by EPR could be envisaged only if the nails could be collected shortly after irradiation. This considerably limits the field of application, considering that the usual delay to identify the accident and to collect and transport the samples is at minimum in the order of days and more likely even weeks. These two major obstacles are the main reasons for the absence of any practical application of nail dosimetry.

Different EPR signals (RIS, MIS and intrinsic) identified in nails are reported in the present paper. The major finding was the identification of a stable RIS, labeled RIS5. A more detailed investigation of this EPR signal is presented. This new finding has made possible to foresee a practical application of human nail dosimetry. Based on the present work, a new protocol of EPR nail dosimetry is proposed. This protocol is designed to identify and estimate a high dose of ionizing radiation to fingers, which is currently the common case of localized irradiation to hands. The protocol has been applied by IRSN for the analysis of nail samples collected from different victims of four radiological accidents that occurred between 2008 and 2012 (Trompier et al. 2013; Romanyukha et al. 2013). In the latest case (Chilca accident), nail dosimetry could be performed in the early management phase which allowed identification of those fingers with the highest dose before the appearance of any clinical signs (IAEA 2012). The data have been decisive in the management of the victims and open the possibility to use human nail EPR dosimetry in radiological accidents, to assist casualties before they become symptomatic. Recent comparison with doses estimated by means of EPR bone dosimetry has also shown the reliability of this newly developed approach (Trompier et al. 2013).

## Materials and methods

Electron paramagnetic resonance (EPR) measurements were performed at two different microwave frequencies (X- and Q-band). The X-band (~9.7 GHz) is the frequency commonly used in dosimetry applications, whereas the Q-band (~34 GHz) was only recently proposed for dosimetry application, with only very few application cases (Romanyukha et al. 2007b; De et al. 2013). The advantages of using the Q-band for nails and calcified tissues (enamel and bones) are the higher sensitivity achieved compared to using the X-band for small mass samples (<10 mg) and the better spectral resolution for these materials (Romanyukha et al. 2011).

In absence of information on registration parameters in the legend of the figures, the following conditions were used. EPR spectra were recorded at room temperature. Measurements were performed with a Bruker EMXplus spectrometer supplied with ER5106QT/W resonator for the Q-band and with a Bruker EMX supplied with SHQ resonator for the X-band. For the Q-band measurements, EPR spectra were recorded with a modulation depth of 0.3 mT and sweep width ranging between 20 and 90 mT. Microwave power was set at 1.23 mW; the scan time was 2.58 s with a time constant of 10.24 ms and the total number of scans ranging between 20 and 50. To investigate the structure of the measured EPR signals in more detail, some measurements were also performed in 90° out of phase and in second harmonic detection mode. All measurements were performed at room temperature. For X-band measurements, EPR spectra were recorded with a modulation depth of 0.3 mT and sweep width ranging from 10 to 50 mT. Microwave power was set at 2 mW, the scan time was 41.96 s with a time constant of 40.96 ms and the total number of scans ranging between 20 and 50.

Nails were collected from finger of human adults (male and female from various ethnicities). Depending of the type of experiment, the delay between the collection and the measurements could range from hours to 4 weeks. When needed, drying of nails was performed in a vacuum dryer with silica gel for 15 h. The heating of samples was done using a glass oven (Büchi, B858 type). Humidification of nails was performed with distilled water.

The irradiations were performed at room temperature with gamma rays sources of ^60^Co and ^137^Cs calibrated in terms of air kerma with electronic equilibrium conditions.

## Results

### Identification of MIS components

**Fig. 1:**
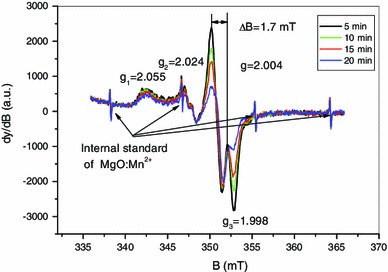
X-band EPR spectra of dry nails mechanically stressed and measured at different times after multiple cuts of the samples

Figure 1 shows the X-band EPR spectra of mechanically stressed human nails measured at room temperature at different times after multiple cuts of the sample. The spectrum of mechanically stressed samples was described by Black and Swarts (2010) as having three components: a doublet at *g* = 2.0044, a singlet at *g* = 2.0055 and a broad anisotropic component originally observed by Chandra and Symons (1987). In Fig. 1, these three components can be clearly observed at *t* = 5 min for the doublet and at *t* = 20 min for the singlet, whereas the broad component does not exhibit a significant intensity decrease as does the doublet, whose intensity is significantly reduced in 20 min. This broad component has been ascribed by Symons et al. (1995) to a σ* species formed on the disulfur bridge formed between two adjacent cysteine groups. In contradiction to this interpretation, the literature on irradiated peptides for which similar features can be observed ascribes the signal to perthiyl (R–SS°) or thiyl (R–S°) radicals (Akasaka 1965; Saxebol and Herskedal 1975; Hadley and Gordy 1975; Bonazzola et al. 1984; Parast et al. 1995). Recent *g* factor calculations of the perthiyl radical by density functional theory (DFT) (Engström et al. 2000) gives similar *g* values (*g*_1_ = 2.063, *g*_2_ = 2.028 *g*_3_ = 2.002) to those observed in Fig. 1, whereas for thiyl radicals and the σ* species, the values are significantly different. Engtröm et al. (2000) calculated a hyperfine coupling of 0.88 mT that is also observed on *g*_2_ in Fig. 1 with a coupling of 0.85 mT. The hyperfine couplings for the other sulfur radicals are very different. This is another argument in favor of the hypothesis of the perthiyl radical. Moreover, regarding the α-keratin structure of nails whose conformation is mainly insured by the disulfur bridges (Farren et al. 2004), the cuts of keratin fibers should lead to the breaking of the bridges. The direct breakage of disulfur bridges can be either homolytic (breakage of the S–S bond) or located on a C–S bond. The EPR spectra of nails grinded in liquid nitrogen and measured at 77 K exhibit the feature of thiyl radicals and a similar feature attributed to perthiyl radicals (Dondi et al. 2010). When the temperature increases, the thiyl component decreases, whereas the perthiyl intensity increases (Dondi et al. 2010). These measurements confirm that both thiyl and perthiyl are formed under mechanical stress. It is known that the reaction of thiyl radicals with thiols leads to the formation of perthiyl radicals and that the reaction rate increases with increasing temperature (Faucitano et al. 2005). All these observations support the hypothesis that the perthiyl radical is the most likely reason for the anisotropic components of the MIS. This component of the MIS is referred to as MIS1.

**Fig. 2:**
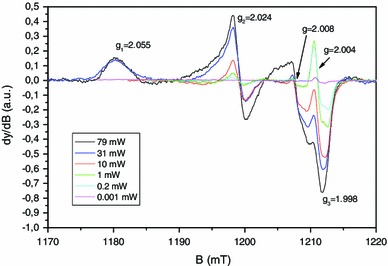
Q-band EPR spectra of mechanically stressed nails measured at different microwave power

**Fig. 3:**
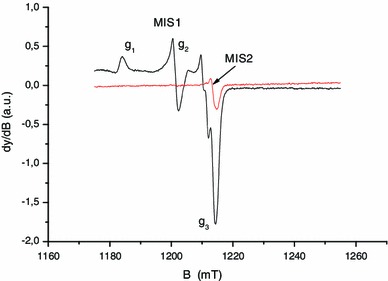
Q-band EPR spectra of mechanically stressed nails, measured in phase (0°) and out of phase (90°). *Black solid line*: 0°, *red solid line*: 90°

The doublet identified by Black and Swarts (2010) was ascribed to carbon-centered radicals produced by the net loss of a hydrogen atom on the α-carbon of the polypeptide backbone that is linked to hydrogen or to a CH_2_R group. The literature on irradiated peptides reveals that such doublets are commonly observed with similar *g* value and hyperfine coupling (see for example Sevilla et al. 1979; Garrison 1987). The coupling with the hydrogen of the carbon-centered radicals can originate from the loss of a glycine amino acid. Glycine is one of the components of the nails keratin. Nevertheless, such doublet can be also observed in irradiated polypeptides that do not contain glycine (Drew and Gordy 1963). In this case, the doublet signal is significantly more unstable than in the case of the glycyl radical. It is known that this non-glycyl radical reacts strongly with dioxygen. In our case, the nails studied in Fig. 1 were dried. In the absence of water, the MIS1 and the singlet do not decay as can be seen in Fig. 1 (Trompier et al. 2007a, b). It is likely that the decay observed for the doublet is not due to the presence of water. Instead, the observed decay of the doublet can be due to a reaction with the dioxygen. In this case, the non-glycyl radical seems to be the most probable reason for the observed doublet. Taking into account hyperconjugation phenomena, the coupling with the CH_2_R group can lead to a doublet as stated by Black and Swarts (2010). The doublet is labeled in our nomenclature as MIS3; the MIS2 is the singlet observed at 2.004. The singlet can be clearly observed by decreasing the microwave power as shown in Fig. 2 or by recording the spectra in the 90° out-of-phase mode (Fig. 3). A component at *g* = 2.008 can also be observed in EPR spectra at Q-band (Fig. 2). This component is affected, as the MIS1, by the microwave power. To the knowledge of the authors, this component of the MIS in nails was not previously described in the literature, contrary to the MIS1, MIS2 and MIS3.

**Fig. 4:**
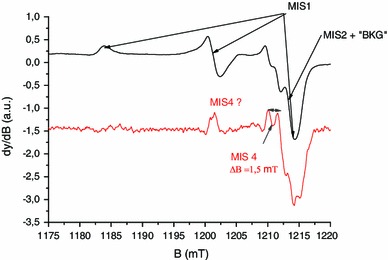
Q-band EPR spectra of mechanically stressed nails measured in the first and second harmonic modes. *Black solid line*: first harmonic, *red solid line*: second harmonic (intensity * 20)

To investigate the structure of this new component labeled MIS4 in more details, Q-band spectra were also recorded in the second harmonic detection mode. As shown in Fig. 4, the measurement in the second harmonic detection mode shows two EPR lines at *g* = 2.008, split by 1.5 mT, possibly connected to a signal at 2.024. One possible hypothesis that could explain this feature is the effect of the sulfinyl radical (RSO°). It has been demonstrated that the sulfinyl radical is the final product of the reaction of thiyl radicals with dioxygen (Sevilla et al. 1987; Becker et al. 1988). This hypothesis is consistent with the fact that the thiyl radical is the primary radical formed under mechanical stress as stated before. Moreover, in irradiated cysteine and saturated in dioxygen, the sulfinyl radical has been identified with the respective *g* values of *g*_1_ = 2.025, *g*_2_ = 2.008 and *g*_3_ = 2.0027, and a hyperfine coupling equal to 1.4 mT that is observed on *g*_2_ (Sevilla et al. 1987). To confirm this hypothesis, nails were grinded under a normal atmosphere and without dioxygen under nitrogen gas.

**Fig. 5:**
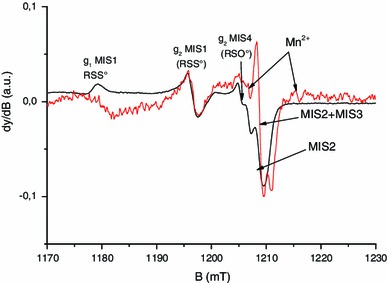
Q-band EPR spectra of nails mechanically stressed under a standard atmosphere and under nitrogen gas. *Black solid line*: standard atmosphere, *red solid line*: N_2_ atmosphere

In Fig. 5, the MIS4 is clearly observed under standard atmosphere conditions, whereas for the experiment under nitrogen gas (without dioxygen) the MIS4 is very weak, as compared to the MIS1 whose intensity is not affected by the modification of the atmosphere. One can also observe the MIS3 component in the nitrogen gas atmosphere, which is not seen under normal atmosphere conditions. This experiment shows that oxygen plays a role in the formation of the MIS4 and in the reduction of the MIS3 as well. Thiyl radicals, as stated before, are primary radicals formed from the homolytic breakage of disulfur bonds by mechanical stress. According to Sevilla et al. (1987), the reaction of thiyl radicals leads to the formation of thiol peroxy radicals (RSSO°) that dissociate in sulfinyl radicals (RSO°). Therefore, it is likely to observe the final products of reaction between thiyl radicals and dioxygen and the sulfinyl at room temperature (Sevilla et al. 1987). To summarize, in addition to the three previously identified components, a fourth one (MIS4) has been observed and identified as a sulfinyl radical.

**Fig. 6:**
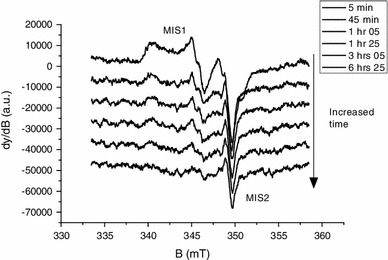
X-band EPR spectra of wet nails mechanically stressed

**Fig. 7:**
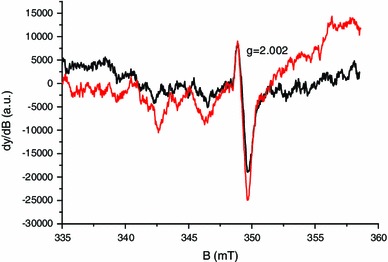
X-band EPR spectra of nails measured 6 h after a first cut (*black solid line*) and 6 h after a second cut (*red solid line*)

**Fig. 8:**
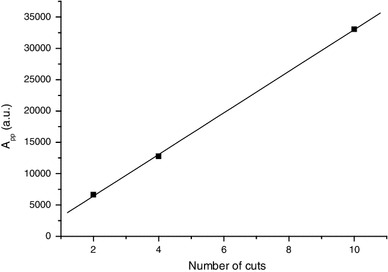
Relationship between the peak-to-peak amplitude (A_pp_) of the stabilized signal measured after cutting and the number of cuts performed in the sample. Measurements were performed at X-band

**Fig. 9:**
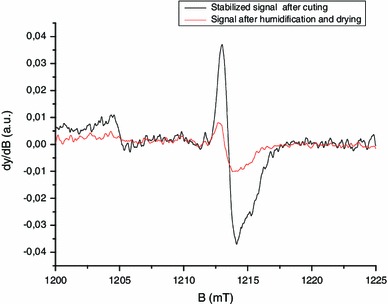
Q-band EPR spectra of a nails measured 6 h after a first cut and after humidification and drying of the nails

Among these four components, the MIS2 is due to the more stable radicals, as shown in Fig. 6. The humidity is known to affect the intensity of the different components of the MIS (Trompier et al. 2007b). Contrary to the spectra shown in Fig. 1, which were obtained using dry nails, spectra from Fig. 6 were obtained using wet nails. The MIS1 intensity in wet nails drastically decreases with time, whereas the singlet (MIS2) intensity slightly decreases. In this experiment, the sample was kept in the cavity under a 2 mW microwave power, resulting in the drying of the sample along the measurement process. After 6 h, the sample can be considered as dried, and accordingly no further signal evolution of the MIS 2 was observed. In several publications the stabilized signal formed by an isotropic singlet was ascribed to an intrinsic signal called “background signal.” However, as shown in Fig. 7, the intensity of the stabilized signal increases with stress, as observed and described in Reyes et al. (2008). It was thus hypothesized in the literature that the so called background signal may also be formed under mechanical stress (Reyes et al. 2008). By plotting the intensity of the stabilized singlet (the peak-to-peak amplitude, *A*_pp_) versus the number of cuts, a linear relation is found (Fig. 8). By applying an additional humidification-drying treatment to the sample used to derive the stabilized signal in Fig. 6, one can observe a significant reduction in the intensity of the singlet, as shown in Fig. 9. This measurement was performed in Q-band to possibly establish the difference between the two spectra shown in the figure. The two signals have similar characteristics in the Q-band. Nevertheless, it is hypothesized that their nature is different or at least their origin is different. The singlet measured after humidification and drying is ascribed to an intrinsic signal. For this signal, we have kept the original name given by Symons et al. (1995) [background signal (BKG)] even if the definition of a background signal is not applicable for this intrinsic signal. The stabilized singlet is assumed to be a mixture of the BKG and the MIS2; the BKG not being eliminated by the humidification. Concerning the exact nature of the free radicals producing these signals, no hypothesis can be formulated at this stage. Further investigations, especially at higher frequency, would be needed to identify the nature of these radicals.

**Fig. 10:**
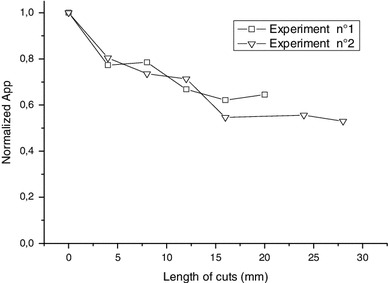
Variation of the normalized peak-to-peak amplitude (*A*_pp_) of the BKG signal (normalized to the signal intensity of the first measurement) after successive cuts of the nails sample. After each cut, the cut edges were humidified to eliminate MIS. For all measurements, the samples have the same geometry in the cavity. The experiment was performed twice. The measurements were performed at X-band

In order to confirm the nature of the so called BKG signal (intrinsic signal), a nail was successively cut, humidified and measured, trying to keep the original geometry of the nail with some tape. The measurements were performed in the X-band mode because the signal intensity is less affected by the sample geometry than in the Q-band mode. Humidification was performed in order to eliminate all the unstable components of the MIS. In Fig. 10, the peak-to-peak amplitude of the singlet measured after each cut and humidification cycle is plotted versus the length of the cuts in the nails samples. Contrary to Fig. 8, the signal intensity (*A*_pp_) decreased after each cycle of cut and humidification. These data show that the signal measured after humidification does not vary with mechanical stress. The humidification was localized on the edge cuts. The decrease in the signal intensity reaches 50 % after 15 mm and then does not vary anymore. According to the previous hypothesis, the signal intensity should have remained constant, but as shown in Reyes et al. (2008), the BKG signal is affected also by the humidification, but in a very different way than the MIS.

**Fig. 11:**
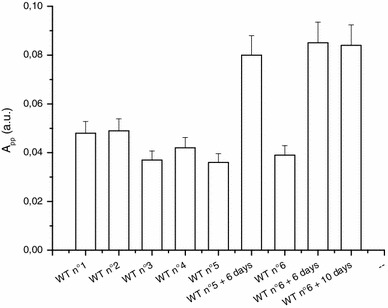
Effect of repeated water treatment of 5 min (WT) and drying in air for 6 or 10 days on the BKG intensity. Measurements were performed at Q-band

**Fig. 12:**
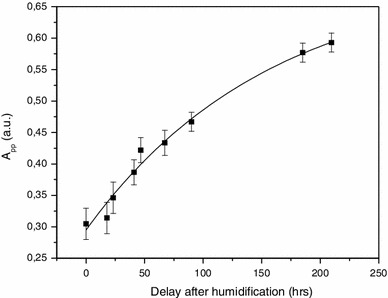
Time dependence of the BKG intensity after a water treatment in distilled water. Measurements were performed at X-band (from Reyes et al. 2008)

The behavior of the BKG signal is illustrated by the data presented in Fig. 11. Even after multiple treatments in distilled water, the BKG does not vary much given the associated uncertainties involved. However, if the nail sample is left to air at ambient air temperature and pressure (ATP), an increase in the signal intensity of the BKG is observed, as seen in Fig. 11 after the water treatment (WT) n°5 i. After a new water treatment (WT n°6), the BKG intensity is reduced to its initial value, while a new delay of 6 days in air at ATP, the BKG intensity increases again in a similar way as after the previous water treatment. A longer delay (>6 days) does not lead to any additional increase in the BKG intensity. The increase in the BKG intensity relatively to the time elapsed after the humidification is shown in Fig. 12 (Reyes et al. 2008). The signal increases continuously until it reaches a saturation level. These data explain the behavior of the BKG intensity presented in Fig. 10. The sample used for this experiment was initially dry. Therefore, by humidifying a local part (cut’s edge) of the nails, in addition to eliminating the MIS components (which are originated at the edge cuts, see Trompier et al. 2009), the BKG signal is also continuously reduced until the whole nail was humidified.

**Fig. 13:**
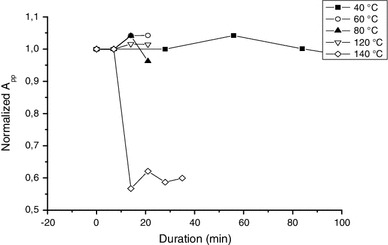
Variation of the BKG intensity (*A*_pp_) upon isothermal annealing treatments. Measurements were performed at X-band

**Fig. 14:**
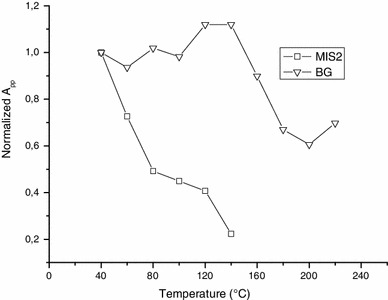
Variation of BKG and MIS2 intensity (*A*_pp_) upon isochronal annealing treatments (ΔT = 20 °C, Δt = 20 min). Measurements were performed at Q-band

Besides the behavior with humidity, the BKG exhibits a very good thermal stability as shown in Fig. 13 with isothermal annealing and in Fig. 14 with isochronal annealing. This aspect is consistent with the hypothesis of having an intrinsic signal.

In summary, four components were identified in the MIS (Table 1). All MIS components can be eliminated with the humidification of nails. The residual signal observed after humidification, the so called background (BKG) signal, is an intrinsic signal and not a MIS. If this BKG signal is thermally very stable, it exhibits an unusual behavior regarding the effect of humidity. After humidification of the nails, the BKG intensity is reduced and 6 days of drying in air restores the initial intensity. The nature of the MIS2 and the BKG remains unidentified at this stage.

**Table 1:** Characteristics of the different types of paramagnetic radicals observed in nails

Origin	Label	EPR parameters	Radical associated	Characteristics
Background	BKG	*g* = 2.004	Not identified	Thermally stable, reversible effect of hydration
Mechanical stress	MIS1	*g*_1_ = 2.055; *g*_2_ = 2.024 (*a*_*H*_ = 0.85 mT); *g*_3_ = 1.998	RSS°	Eliminated with water
	MIS2	*g* = 2.004	Not identified	Eliminated with water
	MIS3	*g* = 2.004 and *a*_*H*_ = 2.0 mT	C° coupled with one proton or two protons with hyperconjugation	Eliminated with O_2_ and water
	MIS4	*g*_1_ = 2.025; *g*_2_ = 2.008 (*a* = 1.5 mT); *g*_3_ = 2.003	RSO°	Eliminated with water
Irradiation	RIS1	*g*_1_ = 2.055; *g*_2_ = 2.023(*a*_*H*_ = 0.85 mT); *g*_3_ = 1.998	RSS°	Formed at high dose, eliminated with water
	RIS2	*g* = 2.005	RSO°_2_	Dominant at dose <1,000 Gy
	RIS3	*g* = 2.004 and *a*_*H*_ = 2.0 mT	C° coupled with one proton or two protons with hyperconjugation	Formed at high dose, eliminated with O_2_ and water
	RIS4	*g*_1_ = 2.025; *g*_2_ = 2.008 (*a* = 1.5 mT); *g*_3_ = 2.003	RSO°	Formed at high dose, eliminated with water
	RIS5	*g* = 2.004	Not identified	Thermally stable and not affected by hydration

### Identification of RIS components

**Fig. 15:**
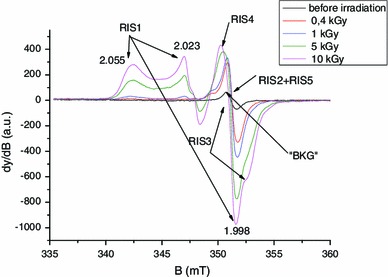
Dependence of the X-band EPR spectra on dose

**Fig. 16:**
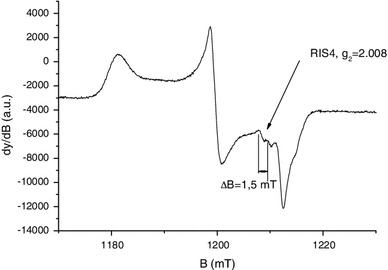
Q-band EPR spectrum of a fingernail irradiated at 10 kGy at room temperature

**Fig. 17:**
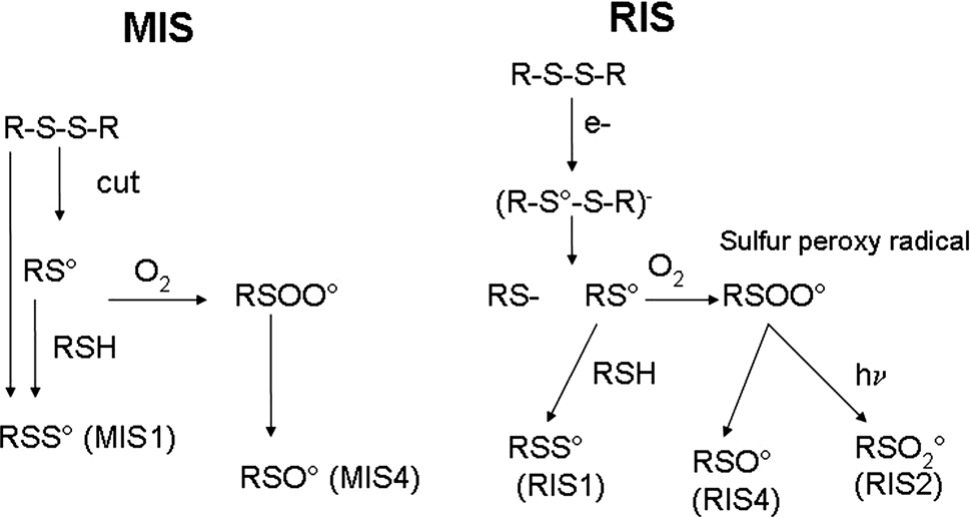
Proposed mechanisms of formation of sulfur radicals in nails by mechanical stress and by irradiation

As shown in Fig. 15, the EPR spectra of irradiated nails significantly change with dose in terms of shape and intensity. In the lower dose range, a singlet is observed, whereas at higher doses, an additional signal appeared with similar features to the MIS1, MIS3 and MIS4. These three RIS components are referred to as RIS1, RIS3 and RIS4. Their characteristics are summarized in Table 1. It is worth noting that the more unstable MIS component (MIS3) has a corresponding RIS at the highest dose. The singlet can be observed at very low doses, while the RIS1 and RIS4 appear after a few hundreds of Gy and the RIS3 after a few thousands of Gy. As for MIS, the interpretation of the EPR spectra at high dose as consisting of three components has already been given in recent literature (Black and Swarts 2010; Wilcox et al. 2010). However, such spectra are currently observed in polypeptide irradiated at a very high dose (see for example Gordy and Miyagawa 1960; Henrikssen et al. 1963) and also in nails and keratinized tissue (horn, silk, wool, etc.) (Gordy and Shields 1958; Shields and Hamrick 1970). The RIS1 and RIS3 components are ascribed to the same type of radicals as those responsible for the MIS1 and MIS3 components, the *g* value and hyperfine coupling being very similar. As for the MIS, an additional component to those previously identified can be observed in Fig. 15. This component, labeled RIS4, can be observed in Fig. 16 in Q-band mode in an EPR spectrum of a fingernail irradiated at 10 kGy. It is interesting to note that different types of stress result in similar EPR features. Although the free radicals ascribed to RIS1, RIS3 and RIS4 are very similar to those ascribed to MIS, the mechanisms of formation are likely to be different (Fig. 17).

**Fig. 18:**
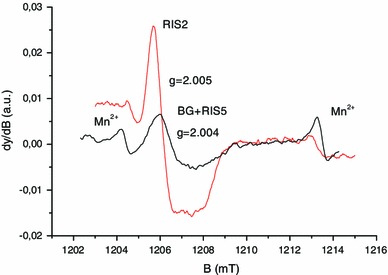
Q-band EPR spectra of fingernails after a 10 Gy irradiation (*red solid line*) and after irradiation followed by 10 min water treatment (*black solid line*)

**Fig. 19:**
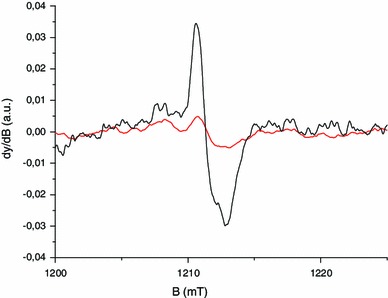
Q-band EPR spectra of fingernails before (*red solid line*) and after irradiation at 60 Gy (*black solid line*)

At lower doses (<1,000 Gy), the spectrum of irradiated nails is dominated by a singlet (RIS2). Whereas in the X-band, it is difficult to differentiate the RIS2 and the BKG, and it is possible to differentiate these two components at Q-band as shown in Fig. 18. Similar results were reported in Romanyukha et al. (2011). Based on the spectrum shown in Fig. 18, the *g* value of the RIS2 (*g* = 2.005) is slightly higher than the *g* value of the BKG and MIS2 (*g* = 2.004). Given this *g* value, it can be hypothesized that the origin of the RIS2 might be the sulfonyl radical (RSO_2_°) that is present in irradiated polypeptide and that shows a similar *g* value and feature (singlet) (Sevilla et al. 1988). However, repeated measurements performed at Q-band before and after irradiation, and after irradiation and water treatment do not show the same *g* shift between the RIS2 and the BKG (Fig. 19). Nevertheless, the sulfonyl radicals remain a possible hypothesis for the origin of these signals; however, further investigations at higher frequencies or irradiation under nitrogen gas need to be performed to further investigate the origin of these signals.

It is worth noting that previous studies on the feasibility of EPR dosimetry with nails were based on the analysis of the RIS2 component. The dose response of this signal was found to be linear up to 300 Gy (Trompier et al. 2009). Nevertheless, the MIS2, as all other MIS or RIS (1, 3 and 4) components, are eliminated by humidification of the nails (Trompier et al. 2007b). Therefore, the applicability of nail dosimetry remains very limited and could be considered only if nails are collected immediately after the irradiation or a few hours later, and if no humidification of nails has occurred.

**Fig. 20:**
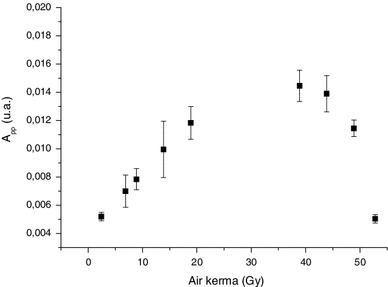
Variation of the intensity of the RIS5 as a function of the delivered dose. Measurements were performed at Q-band

After a water treatment, the signal observed was ascribed to only the BKG (Trompier et al. 2007b, 2009). Nevertheless, if the residual signal measured after irradiation and water treatment is plotted as a function of the irradiation dose (Fig. 20), one can observe a relation between the residual signal intensity and the dose. This means that at low doses and in addition to the RIS2, an additional RIS having similar characteristics as the BKG is identified. This new signal is labeled RIS5. The RIS5 shows a non-conventional dose response because the signal decreases after reaching saturation. Therefore, a given RIS5 intensity is related to two values of dose, which makes it difficult to estimate the dose with conventional approaches such as a calibration curve or the dose additive method. Furthermore, for dosimetry application, it would be difficult to isolate the RIS5 contribution in the total signal, as RIS5 and BKG have the same spectral characteristics. This task is even more difficult given the fact that the BKG intensity varies with humidity. Consequently, it will be necessary to control the hydration state of the investigated nail samples to assess the RIS5 intensity.

**Fig. 21:**
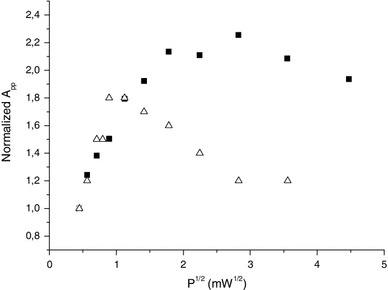
Variation of the signal intensity (*A*_pp_) as a function of the square root of the incident microwave power, for the RIS5 (*solid squares*) and the BKG (*open triangles*). Measurements were performed at X-band

Concerning the origin of these signals, even if the RIS2, BKG and RIS5 have similar EPR characteristics and if they may be of a similar nature, their location and spatial distribution are probably very different. For example, the radicals associated with RIS5 could be located close to hydrophobic amino acids or within the polypeptide helix, which prevent water molecules to react with these radicals, contrary to those associated with RIS2. It is also worth to note that BKG and RIS5 show a very different behavior regarding microwave power as shown in Fig. 21. If both signals are ascribed to the same radical, their spatial distribution maybe very different leading to a very different microwave saturation behavior.

**Fig. 22:**
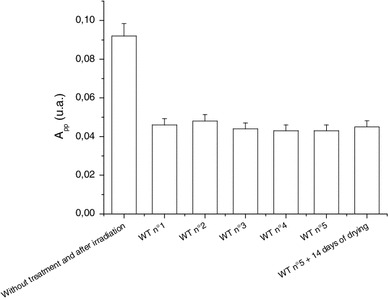
Influence of humidity on the RIS5 intensity determined by multiple water treatment (WT). Measurements were performed at Q-band

**Fig. 23:**
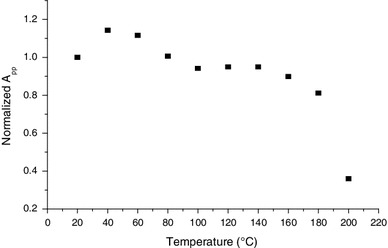
Evolution of the RIS5 intensity (*A*_pp_) upon isochronal annealing treatment (ΔT = 20 °C, Δt = 20 min). Measurements were performed at Q-band

The most important characteristic of RIS5 is that it is still observable after humidification of nails. Figure 22 gives an example of the effect of water on the RIS5 intensity. After irradiation and before any treatment, the signal consists of the BKG, the RIS2 and the RIS5 components. A dose of 40 Gy was applied to maximize the ratio between the RIS5 and the BKG. After the first water treatment, the signal intensity is reduced, the RIS2 being practicably eliminated and the BKG being reduced to its minimum value. Note that the RIS5 intensity is not affected by additional water treatments. One interesting fact is that the drying process does not lead to a similar effect as for the BKG: the intensity of the signal (dominated by the RIS5) remains constant after drying. Moreover, as shown in Fig. 23, the RIS5 exhibits a very good thermal stability similar to the BKG which indicates a high thermal stability at room temperature. Figures 22 and 23 show that, regarding the stability, the RIS5 is a very good candidate for retrospective dosimetry. Nevertheless, the presence of the BKG with a similar signal as the RIS5 and the relatively weak intensity of the RIS5 compared with the BKG are the two main pitfalls that may limit its application in nail dosimetry. For example, a 2 Gy irradiation increases the initial signal intensity of BKG by about 20 %. One can easily understand all the difficulties in performing dosimetry in these conditions: the main difficulty is to differentiate both signals (BKG and RIS5) given the weak RIS5 intensity at doses of a few Gy. Two classical approaches can be taken to differentiate the two signals:

- By using the difference of the signals’ behavior as a function of the applied microwave power, it is possible to isolate the RIS5 component, as it was proposed some years ago for tooth enamel (Ignatiev et al. 1996).
- According to Reyes et al. (2008), the variability of the BKG intensity is relatively weak (about 8 %). It is therefore possible to estimate with a quite good accuracy a reference value for the BKG intensity that could then be subtracted from the signal measured after irradiation. This approach, however, has a main pitfall: it is necessary to perfectly control the hydration state of the investigated nails when measuring the background or the signal after irradiation.

These two approaches can at least in theory be used to estimate the RIS5 intensity. Regarding the calibration of this intensity in terms of radiation dose, another major problem arises as illustrated in Fig. 20. For a given intensity, the RIS5 can correspond to two different doses, because a low dose provides similar signal intensity as a dose of a few tens of Gy. In order to differentiate a low from a high dose, one can irradiate the sample with an additional dose to see whether it will increase the RIS5 intensity or not. Any increase in RIS5 intensity will indicate that the dose under evaluation is below the saturation dose. In the case of a localized irradiation to the hands, some fingers may have received a high dose (few tens of Gy), whereas others may have received a lower dose (a few Gy). Before the appearance of clinical symptoms, it is difficult to localize high-dose spots. Therefore, it seems to be necessary to irradiate the fingernail sample with an additional dose, to decide whether or not the accidental dose was below the saturation dose (Fig. 20). In the case of population triage where the doses are expected in the worst case to be in the order of several Grays, such a post-accident irradiation is not needed if a calibration curve can be established.

**Fig. 24:**
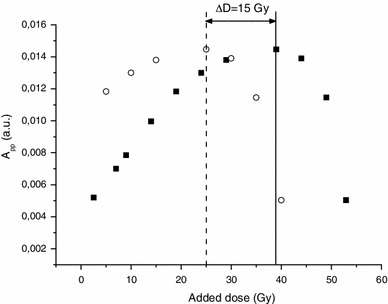
Evolution of the RIS5 intensity (*A*_pp_) regarding multiple irradiation doses for two samples: the first sample was not irradiated (simulation of a non-exposed sample; *solid squares*), while the second sample was irradiated at 15 Gy before the cycle of post-irradiation started (simulation of an accidental exposure of 15 Gy; *open circles*). EPR Measurements were performed at Q-band

Regarding an acute exposure to hands, the signal saturation can at first glance be a problem for dose evaluation; however, one can use it to estimate a dose. Figure 24 shows a way to use this phenomenon to estimate an accidental dose: When performing the post-irradiation in steps of 2 or 5 Gy, one can notice that, due to signal saturation, the value of the added dose is decreased by the value of the dose received by the sample prior to the cycle of post-irradiation. In other words, the higher was the accident dose, the lower will be the added dose to reach the saturation. Therefore, it is possible to deduce the accidental dose by determining the saturation dose on the sample. The difference between the saturation dose of non-exposed nails and exposed nails corresponds to the dose delivered in vivo during the accident (Fig. 24). This method requires knowing the level of saturation for the investigated nails. From our experience, this value is supposed to be identical for a given individual at a given time, but can vary from person to person. On the samples investigated (n = 10), the saturation dose ranges between 28 and 60 Gy with a mean value of 45 ± 8 Gy. The main drawback of this new method is the need of a time-consuming post-irradiation process and the need to identify nails that have not been exposed (hands or feet). Nevertheless, with this method, the dose evaluation does not depend on the evaluation of the BKG intensity as in classical dose estimation methods, which can be a source of error and uncertainty. This new approach in dosimetry is dedicated to the estimation of high doses (>10 Gy) and has been recently applied with success to several cases of severe irradiation accidents with localized irradiations to hands. A description of these applications can be found in Trompier et al. (2013) and Romanyukha et al. (2013). The reliability of this method has been partly demonstrated by comparing the dose estimated with nails with the dose estimated on bone biopsy (phalanx) for several cases (Trompier et al. 2013). Taking into account the dose gradient that might exist in such exposure situations, a good agreement was found between the two assays.

## Conclusion

Several new components in the MIS and the RIS of nails have been identified in this work. Hypotheses regarding their nature were made when possible. It was demonstrated that the BKG is an intrinsic signal that is not induced by mechanical stress. All the components of the MIS and RIS, except one (the RIS5), can be eliminated by humidification of the nails. Due to its stability, the only component that can be used for dosimetric application is the RIS5. Due to its weak intensity and its similarity to the BKG, a dedicated protocol remains to be established. For high-dose application, which is of major interest regarding the number of accident cases with localized severe irradiations to the hands, a new approach in dosimetry using the RIS5 component has been developed and applied. This approach is based on the saturation behavior of the RIS5. More works need to be done, to explore the origin and behavior of all the identified components of the BKG, MIS and RIS. However, in its current state, fingernail dosimetry can be considered a solid dosimetric method that can be used in a complementary way with other dosimetric modalities and future accidental dosimetry using nails as biomarkers of radiation exposure is promising.
